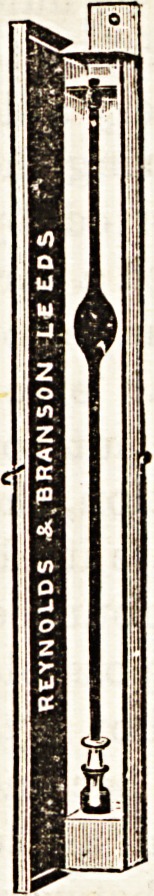# New Appliances and Things Medical

**Published:** 1896-05-16

**Authors:** 


### NEW APPLIANCES AND THINGS MEDICAL.

### ENEMA SYRINGE CUPBOARD.

Messrs. Reynolds and Branson, surgical instrument makers, Briggate, Leeds, to whose practical inventions we often have occasion to call attention, have patented an admirable little "cupboard" made of enamelled tin, for the better preservation of the ordinary Higginson syringe. To keep them properly it is most necessary to suspend them, and at the same time to protect them from dust. Folded in the boxes in which they are bought, they infallibly become cracked and worn. These neat little receptacles provide "the right place" at the very small cost of 1s. 6d., and will be found most useful.

We have received a sample of Morgan's "Hungarian Adhesive Paste," and after having practically tested it, we have no hesitation in saying that we consider it is one of the best of its kind. It possesses strong adhesive qualities, is very clean, inoffensive, and spreads easily, besides being apparently free from the objection which attaches to so many such pastes of hardening and decomposing, unless in constant use. It can be obtained from Messrs. Bemrose and Sons (Limited), Old Bailey, E.C., price 1s. per bottle, with polished cap and brush complete.

## Figures and Tables

**Figure f1:**